# Unintentional Injury Burden in Hong Kong: Results from a Representative Population-Based Survey

**DOI:** 10.3390/ijerph18168826

**Published:** 2021-08-21

**Authors:** Eric Ho Man Tang, Laura Elizabeth Bedford, Esther Yee Tak Yu, Emily Tsui Yee Tse, Weinan Dong, Tingting Wu, Bernard Man Yung Cheung, Carlos King Ho Wong, Cindy Lo Kuen Lam

**Affiliations:** 1 Department of Family Medicine and Primary Care, Li Ka Shing Faculty of Medicine, University of Hong Kong, Hong Kong, China; erichm@hku.hk (E.H.M.T.); lbedford@hku.hk (L.E.B.); emilyht@hku.hk (E.T.Y.T.); dovey@connect.hku.hk (W.D.); wutt1025@connect.hku.hk (T.W.); carlosho@hku.hk (C.K.H.W.); clklam@hku.hk (C.L.K.L.); 2 Department of Medicine, Li Ka Shing Faculty of Medicine, University of Hong Kong, Hong Kong, China; mycheung@hku.hk; 3 Department of Pharmacology and Pharmacy, Li Ka Shing Faculty of Medicine, University of Hong Kong, Hong Kong, China

**Keywords:** unintentional injury, safety, public health, general population

## Abstract

Unintentional injuries are major causes of mortality and morbidity. Although generally perceived as accidents, it is possible to identify those at higher risk and implement appropriate prevention measures. This study aims to investigate the common causes of unintentional injuries and their associated risk factors among a large representative sample. Data of 12,022 individuals who completed the Hong Kong Population Health Survey 2014/15 were extracted. The primary outcome was the prevalence of having unintentional injury(-ies) in the previous 12 months that was severe enough to limit daily activities. Multivariable logistic regression analyses were conducted to identify associations between injuries and sociodemographic, clinical and lifestyle factors. 14.5% of respondents reported episode(s) of unintentional injury in the past 12 months in the population level. The main causes of top three most severe unintentional injuries were sprains (24.0%), falls (19.9%) and being hit/struck (19.6%). 13.2% injury episodes were work-related among the most severe episode. Factors independently associated with significantly higher risks of injury included currently employed, homemaker or student, born in Hong Kong (as compared with immigrants), doctor-diagnosed chronic conditions, harmful alcohol consumption, insufficient sleep, and disturbed sleep. To summarize, unintentional injuries are highly prevalent and associated with harmful drinking, insufficient sleep, and disturbed sleep, which are potential modifiable risk factors for prevention.

## 1. Introduction

Injuries are a major public health problem [1]. It has been estimated that they result in five million deaths each year, accounting for 9% of all deaths worldwide [1]. Injuries are generally categorized and defined as either intentional or unintentional [2]. Intentional injuries are those where it is clear that an individual has purposely harmed themselves or others (e.g., suicide, domestic violence), whereas unintentional injuries can occur by mishap or negligence (e.g., road traffic accidents, drowning, burns, poisoning) [2]. In addition to making up around 75% of all injury-related deaths [1], unintentional injuries tend to be more common among younger and economically productive individuals, thereby resulting in 138 million disability-adjusted life years (DALYs) lost each year [2]. Indeed, those who have experienced an unintentional injury report both short and long-term health consequences that can result in impaired health-related quality of life (HRQoL) [3] and psychological morbidity [4,5] for both the victims and their families [6].

Although generally perceived as accidents, research has shown that unintentional injuries are not random events and that it is possible to implement effective prevention and control measures. The Haddon Matrix is a framework commonly applied to develop injury prevention interventions [7]. This matrix combines the epidemiologic triangle (host, agent, environment) with three levels of prevention [2,7]: (1) pre-injury (e.g., group exercise programs to reduce falls in older people [8], speed cameras to avoid road traffic accidents [9]); (2) injury (e.g., fire escape plan [10]); and (3) post-injury (e.g., first aid training [11]). However, in order to develop successful interventions for specific populations, it is important that epidemiological analyses are conducted to establish the prevalence, causes, and modifiable risk factors [12]. Indeed, such analyses should take place at regular intervals to enable tracking of injury rates and to evaluate the effectiveness of any interventions and control measures [12].

The current study reports on unintentional injury among a random stratified representative sample of the general population of Hong Kong, one of the world’s most densely populated regions. Since 1986, injuries have been among the five leading causes of mortality in Hong Kong [13] and accounted for 1848 deaths in 2019 alone [14]. A population-based descriptive study on injury in Hong Kong was conducted in 2008, which was more than a decade ago [15,16]. The most recent Global Burden of Diseases (GBD), Injuries and Risk Factors Study reported that, between 1990 and 2017, the age-standardized incidence of injuries (both unintentional and intentional) in Hong Kong increased by 58.5% (95% Uncertainty Interval (UI) 53.2% to 64.1%) [17]. Given the high and increasing prevalence of unintentional injuries in Hong Kong, the aims of the current study were to draw a comprehensive picture of the unintentional injuries burden among the general population by (1) determining the common causes, and (2) identifying the sociodemographic, clinical, and lifestyle factors associated with unintentional injuries.

## 2. Materials and Methods

### 2.1. Study Design and Setting

This study was a secondary analysis of data from the Hong Kong Population Health Survey 2014/15 (PHS 2014/15), a cross-sectional population-based study conducted by the Department of Health of the Government of Hong Kong Special Administrative Region (SAR) between December 2014 and October 2015. Systematic replicated sampling was applied to select a representative sample of the land-based non-institutional population (aged ≥15 years), excluding visitors and domestic helpers from outside Hong Kong [18]. In total, 7205 households were contacted and 5435 agreed to participate (household response rate: 75.4%). Among the recruited households, 12,022 individuals completed the face-to-face interviews, which consisted of a series of items covering sociodemographic information, health-related quality of life (HRQoL), health status, lifestyle, unintentional injury, preventive health practices, and healthcare utilization.

### 2.2. Outcomes and Predictor Variables

The primary outcome was self-reported unintentional injury in the past 12 months that was severe enough to limit daily activities. We also extracted data on the cause of each of the three most severe injuries (if applicable), the part(s) of the body that was injured in the most severe unintentional injury episode, the location where the most severe unintentional injury occurred, and whether the injury was work- or sport-related.

Predictor variables as potential risk factors of unintentional injury comprised socio-demographic information, clinical, and lifestyle factors. For sociodemographic variables, this included gender (male, female), age groups for different life stages (<35 years for young adults, 35–54 years for middle-aged people, 55–74 years for older adults, and 75 years or above for elderlies), employment status (employed in an office-based/managerial/administrative capacity, employed in a manual/physical work role, unemployed, retired, homemaker, and student), marital status (never married, married, divorced/separated, and widowed), immigrant background (born in Hong Kong, immigrants living in Hong Kong for ≥seven years who are eligible for permanent residency, and immigrants living in Hong Kong for <seven years), educational background (no schooling/pre-primary, primary, secondary, and tertiary) and monthly household income. At the time of data collection, the Hong Kong median monthly household income was HK$24,500. Therefore, for the current analyses, income was categorized as follows: (1) <50% of the median, (2) ≥50% of the median to median, and (3) ≥the median.

Clinical variables assessed as potential risk factors were self-reported doctor-diagnosed chronic diseases (excluding mental health conditions) and doctor-diagnosed mental health conditions.

Finally, a number of lifestyle variables were included: (1) sleep hours (<six hours, six to eight hours, and ≥nine hours of sleep on average per day to represent insufficient, normal and long sleep hours [19,20]), (2) any sleep disturbance (difficulty initiating sleep, intermittent awakenings during the night, and/or early morning awakening) ≥ three times per week, (3) physical activity level (sufficient level defined by performing at least 150 min moderate-intensity, at least 75 min vigorous-intensity physical exercise per week, or an equivalent combination of moderate- and vigorous-intensity physical activity achieving at least 600 MET-minutes, as recommended by the World Health Organization (WHO) [21]), (4) smoking status (never, current, and former), and (5) alcohol consumption (never, former, non-harmful current, and harmful current). Harmful alcohol consumption was defined as an Alcohol Use Disorders Identification Test (AUDIT) score of ≥8 [22].

### 2.3. Statistical Analysis

Descriptive statistics (e.g., number of respondents (*N*) and proportion (%)) were used to describe the characteristics of respondents. The significance of differences between groups (with unintentional injury, without unintentional injury) was assessed by non-parametric chi-square test. In addition to the results in sample level, weighting factors compiled by the Department of Health of the Government of Hong Kong SAR were applied to estimate the descriptive results in population level [18]. After applying the population weighting, the distribution of gender and age group is the same as the land-based non-institutional population of Hong Kong in the second quarter of 2015 [18].

Multivariable logistic regressions were used to assess the adjusted effect of socio-demographic, clinical, and lifestyle factors on the likelihood of reporting an unintentional injury in sample level. Following this, subgroup analyses on the association between work- or sport-related unintentional injury as the most severe injury episode and respondents’ factor were also assessed, adjusting for socio-demographic, clinical, and lifestyle factors. Interaction between sleep hours, any sleep disturbances, and alcohol consumption were also considered in the multivariable logistic regression. Yet, none of the interaction terms had a significant effect on likelihood of injury and they were dropped in the final logistic regression model. A small number of respondents (*N* = 64) were excluded from the multivariable logistic regression analyses because of missing data on household income (*N* = 36) or having invalid occupational status data as a result of receiving financial assistance from the government or being unable to work due to a long-term illness, disability, mental health problem, or occupational injury (*N* = 28). Multicollinearity between independent variables was assessed by variation inflation factors. As all variation inflation factors were <10, the effect of multicollinearity is said to be small.

Statistical analyses were performed using Stata version 13.0 (StataCorp LP. College Station, TX, USA). All tests of significance were two-tailed with a *p*-value of <0.05 considered to be statistically significant.

### 2.4. Ethical Approval

As this was a secondary analysis of open-source, anonymized government data, ethical approval from the Institutional Review Board of the University of Hong Kong/Hospital Authority Hong Kong West Cluster was waived.

## 3. Results

### 3.1. Respondents

12,022 respondents were sampled in PHS 2014/15. After applying the population weighting, just over half were female (52.4%), those aged between 35 and 54 years formed the largest proportion among the age groups (36.3%) and the majority were employed (58.3%) (Table 1). For clinical factors, 39.1% reported a doctor-diagnosed chronic condition with the most common being hypertension (17.8%), high blood cholesterol (14.4%), and eye diseases (8.7%). It was also found that 1.6% reported a doctor-diagnosed mental health condition. For lifestyle, 12.7% had insufficient physical activity, 27.1% were current or ex-smokers of cigarettes, 58.0% were non-harmful current drinkers, 9.7% slept less than 6 h per day and 10.5% had sleep disturbances ≥ three times per week.

### 3.2. Prevalence and Risk Factors of Unintentional Injuries

The preceding 12-month prevalence of any unintentional injury that was severe enough to limit daily activities in Hong Kong population was 14.5%. Based on the sample level, respondents reporting an unintentional injury were more likely to be employed in an office-based/managerial/administrative capacity, single, born in Hong Kong (as compared to immigrants), educated to university level or above, have a doctor-diagnosed chronic condition, a mental health condition, insufficient physical activity, be a harmful current drinker, have less than six hours of sleep, and experience sleep disturbances (all *p* < 0.05) (Table 1).

When the doctor-diagnosed chronic conditions were analyzed separately, those with an unintentional injury were more likely to have: stroke, respiratory disease, diseases of the ears/nose/throat, thyroid disease, kidney disease, liver, stomach and intestinal diseases, congenital blood diseases, musculoskeletal diseases, skin diseases, eye disease or hearing problem (all *p* < 0.05). There were no statistically significant differences between groups in terms of gender, monthly household income, or smoking status (Table 1).

As shown in Table 2, the main causes of the top three most severe injuries were sprains (24.0%), falls (19.9%), being hit/struck (19.6%), cutting/piercing (15.8%), and sport (12.8%). Among the most severe injury episode, the majority of injuries were to the distal limbs: wrist/hand/finger (28.6%) followed by ankle/foot/toe (24.0%) and knee/lower leg (24.0%) (Table 3). Unintentional injuries most commonly occurred in the home (28.5%), followed by sport/athletics area (17.2%) and transport area (16.7%). 13.2% of injuries were work-related.

The results of the multivariable analysis in sample level, where each variable is adjusted for all other variables included in the model, are presented in Table 4. For socio-demographics, respondents who were employed (as both office-based and physical work), a homemaker, or student had significantly higher odds of unintentional injury (OR 1.61 [*p* < 0.001], 1.56 [*p* < 0.001], 1.30 [*p* = 0.030], and 1.72 [*p* = 0.001], respectively), than retirees. Those born in Hong Kong had a 49% higher chance of injury than immigrants living in Hong Kong for < seven years (OR 1.49, *p* = 0.007). When compared to those with secondary level education, respondents with no schooling/pre-primary or tertiary education had significantly higher odds of reporting an injury (no schooling/pre-primary: OR 1.38 [*p* = 0.029], tertiary: OR 1.17 [*p* = 0.021]).

Respondents with the following doctor-diagnosed chronic diseases had increased odds of reporting an injury than those without such conditions: respiratory diseases (OR 1.63, *p* < 0.001); diseases of the ears, nose or throat (OR 2.20, *p* < 0.001); liver, stomach and intestinal diseases (OR 1.59, *p* = 0.001); congenital blood diseases (OR 1.58, *p* = 0.007); musculoskeletal diseases (OR 1.31, *p* = 0.040); skin diseases (OR 1.51, *p* = 0.001); eye diseases (OR 1.43, *p* < 0.001); and hearing problems (OR 1.41, *p* = 0.037).

In terms of lifestyle, it was found that respondents who reported harmful alcohol consumption had greater odds of injury than those who reported never drinking alcohol (OR 1.52, *p* = 0.005). Respondents who reported a sleep disturbance ≥3 times a week had a 68% increase in the odds of injury than those who did not report sleep disturbances (OR 1.68, *p* < 0.001). Finally, respondents with <6 h of sleep per day had a 38% increase in the odds of an unintentional injury than respondents with 6 to 8 h of sleep (OR 1.38, *p* < 0.001). Interaction between sleep hours, any sleep disturbances and alcohol consumption were insignificant to the likelihood of unintentional injury, and therefore were not included in the final logistic regression model. The results of interaction between sleep and alcohol consumption are displayed in Supplementary Table S1.

Table 5 shows the results of the subgroup analysis where the adjusted effect of sociodemographic characteristics and lifestyle factors on likelihood of sustaining a work-related or sport-related unintentional injury was explored. The following factors were significantly associated with higher odds of reporting a work-related injury: employed (manual/physical work) (OR 2.51, *p* < 0.001), born in Hong Kong (OR 2.25, *p* < 0.001), immigrant in Hong Kong for ≥7 years (OR 3.43, *p* = 0.010), primary (OR 2.40, *p* = 0.019) or secondary education (OR 2.37, *p* < 0.001), sufficient physical activity level (OR 3.02, *p* = 0.001) and never smoker (OR 2.24, *p* = 0.005).

A number of factors were significantly associated with increased odds of having a sport-related injury: male gender (OR 3.19, *p* < 0.001), younger age (<35 [OR 2.76, *p* = 0.018] or 35–54 [OR 2.47, *p* = 0.016] years), employed (office-based/managerial/administrative) (OR 3.17, *p* = 0.001) or student (OR 8.26, *p* < 0.001), insufficient physical activity level (OR 2.74, *p* = 0.006), never (OR 2.33, *p* = 0.007) and former smoker (OR 2.41, *p* = 0.015), and harmful current drinker (OR 2.37, *p* = 0.038).

## 4. Discussion

The preceding 12-month prevalence of any unintentional injury that was severe enough to limit daily activities was 14.5%. Based on the estimate of the population at the second quarter of 2015, this corresponds to 879,600 individuals (aged ≥15 years). This finding is concerning, as it represents a significant increase from the 12-month prevalence of unintentional injuries reported in the 2008 population-based injury survey (6.2%) (representing 415,200 individuals), despite selected age sampling were difference in the two surveys [15,16]. This trend is also in line with the recent GBD, Injuries and Risk Factors Study where, between 1990 and 2017, an increase in the age-standardized incidence rates of unintentional injuries and transport injuries was found across the 31 provinces in Mainland China, Hong Kong SAR, Macao SAR, and Taiwan (unintentional injuries: 55.5% increase (95% uncertainty interval [UI] 50.7% to 60.3%), transport injuries: 82.2% increase (95% UI 73.7% to 89.9%) [17] with a 58.5% increase in all injuries found in Hong Kong (95% UI 53.2% to 64.1%)) [17]. The years lived with disability (YLD) due to injury also rose in Hong Kong by 53.1% (95% UI 48.5% to 57.3%). These findings are troubling, especially given that the global age-standardized incidence for all injuries had declined by 0.9% between 1990 and 2017 (95% UI −2.3% to 0.6%) [23]. However, it is important to note that Hong Kong is a densely populated metropolitan region with a hilly landscape, crowded streets, heavy traffic, and small square footage of living space per person, all of which raise the risk of injuries. It should also be noted that age-standardized disability-adjusted life years (DALY) and mortality rates related to injury over the same period decreased in Hong Kong by 17.7% (95% UI −27.8% to −6.8%) and 36.9% (95% UI (−47.4% to −24.8%), respectively [17]. Taken together, these results suggest that post-injury intervention (e.g., first aid, healthcare) has greatly improved, however, pre-injury and injury prevention should continue to be a priority for Hong Kong.

### 4.1. Type and Causes of Unintentional Injuries

More than one-tenth of unintentional injuries were work-related (13.2%), with the main causes being sprains, falls, and being hit/struck. As a small geographical region with a large and growing population, there is increasing renovation and construction of buildings (especially high-rise buildings) to accommodate residents, workplaces, and facilities in Hong Kong. A recently published study investigated trends of construction occupational accidents in Hong Kong [24]. It was found that the proportion of construction workers had increased by 89.5% between 2011 (*n* = 62,635) and 2017 (*n* = 118,674). Although injury incidence rates declined from 5.1 per 62,635 workers in 2011 to 3.5 per 118,674 workers in 2017, there was a corresponding increase in the proportion of injuries reported (28%) with ‘slip, trip or fall on same level’ being the most common cause of injury [24]. 

Furthermore, using a computer and sitting in a static posture for long periods of time raises the risk of suffering a repetitive strain finger and wrist injury (RSI). It has been reported that RSIs are common occupational health problems in Hong Kong [25]. This is not surprising given that a large number of workers use computers and work for long hours at a mean of 42 h per week, which is above the global average [26]. In term of compensation, it is less likely for an employee in Hong Kong to intentionally injure himself/herself in order to claim compensation. According to the Employees’ Compensation Ordinance, all cases should be reported to the Labour Department of the Government of Hong Kong SAR within 14 days from the occurrence [27]. The severity of the injury is assessed by a registered medical practitioner with clinical judgement and sick leave granted to the employee if necessary. The employer is then required to pay for the compensation, which is up to 80% of the income loss of the employee during the period of incapacity up to 24 months afterwards. This allows the employee to receive medical treatment and rehabilitation. In this case, there is no extra benefit for an employee to have an injury, suggesting that the injuries identified in our analysis were most likely unintentional.

Finally, it is important to note that 19.9% of unintentional injuries were caused by falls, which reflects an aging population [28] and deserves more public health attention as falls have high morbidity and mortality in elderly people.

### 4.2. Risk Factors for Unintentional Injuries

For sociodemographic factors, respondents who were employed, a homemaker, or a student were significantly more likely to report an injury than retirees. As stated above, those employed could be at higher risk due to long hours using computers or construction accidents. Homemakers are undertaking potentially hazardous activities, including care of children and elderly people and household maintenance duties (e.g., cooking, cleaning) and students could be at higher risk due to sport participation [29] or spending long hours at their computer doing course work. Those born in Hong Kong were more likely to report an injury than those living in Hong Kong for less than seven years but further research is required to confirm this. It was also found that no schooling/pre-primary education and tertiary education were significantly associated with higher odds of injury. A potential explanation is that those with a lower education level are more likely to be employed in positions that carry a higher risk of work-related injury, such as factory work, hospitality, and construction. On the other hand, those with tertiary education may be more likely to have injuries related to computer work or participation in sport.

A number of doctor-diagnosed chronic conditions were found to be positively associated with unintentional injuries (e.g., diseases of the ears/nose/throat, musculoskeletal conditions). It is likely that symptoms caused by such conditions (e.g., fatigue, postural hypotension, muscle weakness, sensory impairment), their impact on physical fitness, and the side effects from medications (e.g., drowsiness, dizziness, hypoglycemia) could all increase the risk of injury. It is important that patients with these conditions are specifically counselled on their increased risk of injury and advised on preventive measures.

In terms of lifestyle factors, any reported sleep disturbance ≥three times per week was the lifestyle variable most strongly associated with unintentional injury. Insufficient sleep was also found to be significantly associated with injury. This finding is consistent with the results of previous studies [30,31,32,33], with one systematic review and meta-analysis reporting that 13% of occupational injuries were explained by sleep issues alone [31]. Indeed, sleep is an essential physiological function and sleep problems can result in reduced attention span and concentration, as well as daytime drowsiness, all of which increase the likelihood of errors and injuries. Hong Kong is known as a sleep-deprived region and our results showed that sleep problems are common with 9.9% of respondents reporting insufficient sleep and 10.7% reporting sleep disturbances ≥ three times per week (Table 1). However, public health promotion of sufficient and good quality sleep is relatively lacking when compared with promotion of physical activity and healthy eating. It was also found that harmful current drinking was significantly associated with an increased risk of injury, including sport-related injury. Indeed, previous research has identified strong associations between alcohol consumption and injury due to the impairments caused by intoxication (e.g., loss of co-ordination, drowsiness) [34]. 

### 4.3. Strengths and Limitations

The data included in this study are from a large replicative sample of the general population, and quality control was rigorous, which increases generalizability. We were able to identify specific high-risk groups and a range of potentially modifiable clinical and lifestyle risk factors.

However, our results have some limitations. First, the study was cross-sectional, which carries the possibility of reverse causation. In addition, injuries were self-reported and their severity could have been underestimated or overestimated depending on the respondent’s self-assessment. Moreover, fatal unintentional injuries were not captured. It is also important to note that domestic helpers comprise around 5% of the local population and undertake caring and housekeeping duties, which could put them at higher risk of injury. It is possible that their exclusion from the PHS 2014/15 could lead to the underestimation of the overall injury prevalence rate for Hong Kong. Lastly, the use of drugs among respondents was not captured in PHS 2014/15, so we were unable to explore associations between drug use and injury.

### 4.4. Implications for Future Research

Future studies should apply prospective methodologies to identify causal associations and examine potential interactions among risk factors. Further research should also seek to develop and test injury prevention interventions. Digital interventions (e.g., virtual-reality programs, video demonstrations) may have the greatest potential since they can be made widely available and accessed at any time. Indeed, two recently published reviews reported that digital interventions are effective at reducing unintentional injury rates [35,36]. Such interventions may also be more appealing to young adults who are a high-risk group for injuries.

### 4.5. Public Health and Clinical Implications

Tailored public health campaigns should be delivered to raise awareness of injury prevention strategies among high-risk groups. For example, tips to prevent computer-related RSIs in workplaces such as including regular breaks, adopting good posture, and exercising/stretching frequently. Educational interventions and campaigns should also continue to target modifiable risk factors, such as attention to the quantity and quality of sleep, and abstinence from harmful alcohol consumption. It is important that injury prevention is included within all strategies for health and wellbeing. In terms of clinical implications, healthcare professionals have a key role to play in unintentional injury prevention. For example, anticipatory advice to patients with chronic diseases regarding the appropriate precautions to take, as well as general home safety advice including appropriate precautions if working at a great height, or otherwise tips like ensuring adequate lighting, removing loose rugs, and installing handrails for toilets and the bath/shower.

## 5. Conclusions

Unintentional injuries are highly prevalent in Hong Kong’s population. The majority of injuries were work-related and caused by sprains, falls, and being hit/struck. Insufficient and poor quality sleep, and harmful drinking are potential modifiable risk factors for pre-injury prevention. It was especially concerning to find that the prevalence of injuries in Hong Kong has increased over time, which is in line with the trend in prevalence rates across Mainland China, Macao SAR, and Taiwan. There is therefore a pressing need to raise public awareness of unintentional injuries and to develop effective interventions targeting the most common causes of injuries and high-risk groups.

## Figures and Tables

**Table 1:** Characteristics of study participants by whether or not they experienced an unintentional injury in the previous 12 months.

		Sample Level							Projected Population for the Second Quarter of 2015 ^¶^					
		Total (*N* = 12,022)		With Unintentional Injury (*N* = 1737, 14.4%)		Without Unintentional Injury (*N* = 10,285, 85.6%)		*p*-Value	Total (*N* = 6,080,200)		With Unintentional Injury (*N* = 879,557, 14.5%)		Without Unintentional Injury (*N* = 5,200,643, 85.5%)	
		*N*	%	*N*	%	*N*	%		*N*	%	*N*	%	*N*	%
**Gender**								0.082						
	Male	5665	47.1%	852	49.1%	4813	46.8%		2,895,200	47.6%	437,730	49.8%	2,457,470	47.3%
	Female	6357	52.9%	885	51.0%	5472	53.2%		3,185,000	52.4%	441,827	50.2%	2,743,173	52.8%
**Age group**								<0.001 *						
	<35	3437	28.6%	578	33.3%	2859	27.8%		1,763,000	29.0%	295,338	33.6%	1,467,662	28.2%
	35–54	4261	35.4%	584	33.6%	3677	35.8%		2,204,200	36.3%	303,886	34.6%	1,900,314	36.5%
	55–74	3308	27.5%	428	24.6%	2880	28.0%		1,629,500	26.8%	210,231	23.9%	1,419,269	27.3%
	75 or above	1016	8.5%	147	8.5%	869	8.5%		483,500	8.0%	70,102	8.0%	413,398	8.0%
**Employment status**								<0.001 *						
	Employed (office-based/managerial/administrative)	4935	41.1%	774	44.6%	4161	40.5%		2,610,058	42.9%	409,366	46.5%	2,200,692	42.3%
	Employed (manual/physical work)	1877	15.6%	273	15.7%	1604	15.6%		935,067	15.4%	135,884	15.5%	799,183	15.4%
	Unemployed	373	3.1%	47	2.7%	326	3.2%		188,505	3.1%	23,768	2.7%	164,737	3.2%
	Retired	2244	18.7%	275	15.8%	1969	19.1%		1,076,285	17.7%	132,084	15.0%	944,202	18.2%
	Homemaker	1623	13.5%	205	11.8%	1418	13.8%		800,980	13.2%	100,315	11.4%	700,665	13.5%
	Student	928	7.7%	158	9.1%	770	7.5%		448,590	7.4%	75,655	8.6%	372,935	7.2%
	Other	42	0.4%	5	0.3%	37	0.4%		20,714	0.3%	2,484	0.3%	18,229	0.4%
**Marital status**								<0.001 *						
	Single	3575	29.7%	586	33.7%	2989	29.1%		1,810,369	29.8%	296,301	33.7%	1,514,068	29.1%
	Married	7159	59.6%	952	54.8%	6207	60.4%		3,648,378	60.0%	487,539	55.4%	3,160,838	60.8%
	Divorced/Separated	528	4.4%	82	4.7%	446	4.3%		256,233	4.2%	38,707	4.4%	217,527	4.2%
	Widowed	760	6.3%	117	6.7%	643	6.3%		365,220	6.0%	57,010	6.5%	308,210	5.9%
**Immigrant background**								<0.001 *						
	Born in Hong Kong	7296	60.7%	1134	65.3%	6162	59.9%		3,741,483	61.5%	582,376	66.2%	3,159,107	60.7%
	Immigrant has lived in Hong Kong for 7 years or more	4215	35.1%	548	31.6%	3667	35.7%		2,056,389	33.8%	267,740	30.4%	1,788,649	34.4%
	Immigrant has lived in Hong Kong for less than 7 years	511	4.3%	55	3.2%	456	4.4%		282,328	4.6%	29,441	3.4%	252,887	4.9%
**Educational background**								<0.001 *						
	No schooling/Pre-primary	559	4.7%	92	5.3%	467	4.5%		267,117	4.4%	44,296	5.0%	222,821	4.3%
	Primary	1997	16.6%	264	15.2%	1733	16.9%		937,393	15.4%	123,876	14.1%	813,517	15.6%
	Secondary	6276	52.2%	854	49.2%	5422	52.7%		3,154,145	51.9%	426,853	48.5%	2,727,292	52.4%
	University or above	3190	26.5%	527	30.3%	2663	25.9%		1,721,545	28.3%	284,531	32.4%	1,437,014	27.6%
**Monthly household income**								0.804						
	Below 12,250 HKD	2250	18.7%	318	18.3%	1932	18.8%		1,079,607	17.8%	151,972	17.3%	927,635	17.8%
	12,250–24,499 HKD	2959	24.6%	433	24.9%	2526	24.6%		1,453,202	23.9%	212,227	24.1%	1,240,975	23.9%
	24,500 or above HKD	6777	56.4%	979	56.4%	5798	56.4%		3,529,926	58.1%	512,155	58.2%	3,017,771	58.0%
	Unanswered	36	0.3%	7	0.4%	29	0.3%		17,465	0.3%	3,203	0.4%	14,262	0.3%
**Doctor-diagnosed chronic conditions (excluding mental illness)**								<0.001 *						
	Yes	4795	39.9%	801	46.1%	3994	38.8%		2,376,834	39.1%	399,639	45.4%	1,977,195	38.0%
	No	7227	60.1%	936	53.9%	6291	61.2%		3,703,366	60.9%	479,918	54.6%	3,223,448	62.0%
**Type of chronic condition (excluding mental illness)**														
	Cancer	188	1.6%	24	1.4%	164	1.6%	0.508	90,409	1.5%	11,953	1.4%	78,456	1.5%
	Stroke	179	1.5%	36	2.1%	143	1.4%	0.030 *	84,801	1.4%	16,974	1.9%	67,827	1.3%
	Coronary heart disease	261	2.2%	42	2.4%	219	2.1%	0.445	126,564	2.1%	20,061	2.3%	106,503	2.1%
	Respiratory disease	307	2.6%	80	4.6%	227	2.2%	<0.001 *	152,170	2.5%	38,839	4.4%	113,331	2.2%
	Neurological disease	26	0.2%	5	0.3%	21	0.2%	0.487	12,151	0.2%	2,433	0.3%	9,717	0.2%
	Diseases of the ears/nose/throat	268	2.2%	86	5.0%	182	1.8%	<0.001 *	133,237	2.2%	43,371	4.9%	89,866	1.7%
	Thyroid disease	255	2.1%	50	2.9%	205	2.0%	0.018 *	124,522	2.0%	25,091	2.9%	99,431	1.9%
	Kidney disease	81	0.7%	21	1.2%	60	0.6%	0.003 *	39,327	0.6%	10,677	1.2%	28,650	0.6%
	Liver, stomach and intestinal diseases	321	2.7%	76	4.4%	245	2.4%	<0.001 *	162,151	2.7%	38,503	4.4%	123,648	2.4%
	Congenital blood diseases	211	1.8%	54	3.1%	157	1.5%	<0.001 *	105,302	1.7%	27,054	3.1%	78,247	1.5%
	Musculoskeletal diseases	424	3.5%	94	5.4%	330	3.2%	<0.001 *	206,724	3.4%	46,615	5.3%	160,109	3.1%
	Immune diseases	62	0.5%	8	0.5%	54	0.5%	0.729	30,065	0.5%	3,845	0.4%	26,220	0.5%
	Skin diseases	368	3.1%	96	5.5%	272	2.6%	<0.001 *	186,460	3.1%	48,707	5.5%	137,752	2.7%
	High blood cholesterol	1748	14.5%	268	15.4%	1480	14.4%	0.256	873,170	14.4%	134,208	15.3%	738,962	14.2%
	Hypertension	2232	18.6%	324	18.7%	1908	18.6%	0.920	1,079,450	17.8%	155,657	17.7%	923,793	17.8%
	Diabetes	694	5.8%	101	5.8%	593	5.8%	0.936	332,739	5.5%	48,456	5.5%	284,283	5.5%
	Eye disease	1100	9.2%	204	11.7%	896	8.7%	<0.001 *	529,707	8.7%	98,526	11.2%	431,181	8.3%
	Hearing problem	274	2.3%	62	3.6%	212	2.1%	<0.001 *	131,116	2.2%	29,479	3.4%	101,637	2.0%
**Doctor-diagnosed mental health condition**								0.016 *						
	Yes	201	1.7%	41	2.4%	160	1.6%		98,538	1.6%	20,215	2.3%	78,323	1.5%
	No	11,821	98.3%	1696	97.6%	10,125	98.4%		5,981,662	98.4%	859,342	97.7%	5,122,320	98.5%
**Physical activity (sufficient as recommended by WHO) ^†^**								0.003 *						
	Sufficient	10,480	87.2%	1476	85.0%	9004	87.5%		5,305,260	87.3%	749,814	85.2%	4,555,446	87.6%
	Insufficient	1542	12.8%	261	15.0%	1281	12.5%		774,940	12.7%	129,743	14.8%	645,197	12.4%
**Smoking status**								0.195						
	Never smoker	8761	72.9%	1237	71.2%	7524	73.2%		4,431,769	72.9%	625,699	71.1%	3,806,069	73.2%
	Former smoker	1496	12.4%	236	13.6%	1260	12.3%		748,128	12.3%	118,066	13.4%	630,062	12.1%
	Current smoker	1765	14.7%	264	15.2%	1501	14.6%		900,303	14.8%	135,791	15.4%	764,512	14.7%
**Alcohol consumption**								0.001 *						
	Never drinker	2710	22.5%	362	20.8%	2348	22.8%		1,327,721	21.8%	178,152	20.3%	1,149,568	22.1%
	Former drinker	2041	17.0%	274	15.8%	1767	17.2%		1,016,816	16.7%	135,344	15.4%	881,472	17.0%
	Non-harmful current drinker ^‡^	6859	57.1%	1018	58.6%	5841	56.8%		3,523,179	58.0%	522,801	59.4%	3,000,377	57.7%
	Harmful current drinker ^‡^	412	3.4%	83	4.8%	329	3.2%		212,485	3.5%	43,260	4.9%	169,225	3.3%
**Sleep hours**								<0.001 *						
	<6 h	1186	9.9%	252	14.5%	934	9.1%		588,199	9.7%	125,071	14.2%	463,129	8.9%
	6–8 h	10,109	84.1%	1374	79.1%	8735	84.9%		5,133,690	84.4%	699,990	79.6%	4,433,700	85.3%
	≥9 h	727	6.1%	111	6.4%	616	6.0%		358,311	5.9%	54,497	6.2%	303,814	5.8%
**Any sleep disturbance ^§^**								<0.001 *						
	Yes	1289	10.7%	303	17.4%	986	9.6%		640,132	10.5%	149,708	17.0%	490,425	9.4%
	No	10,733	89.3%	1434	82.6%	9299	90.4%		5,440,068	89.5%	729,849	83.0%	4,710,219	90.6%

Note: * Significant at 0.05 level by Chi-square test. ^†^ The estimates of physical activity level were different from the report in the Population Health Survey 2014/15, which only included respondents aged 18 or above for the calculation of physical activity level. ^‡^ Harmful drinking is defined as an Alcohol Use Disorders Identification Test (AUDIT) score ≥8. ^§^ Sleep disturbance includes having difficulty in falling asleep, maintaining sleep, and early morning awakenings. ^¶^ Population weighting allocated by age group and gender of survey respondents was applied to sample level to estimate the results with respect to the land-based non-institutional population of Hong Kong in the second quarter of 2015.

**Table 2:** The main causes of the top three most severe unintentional injuries over the previous 12 months.

Main Cause of Injury	Sample Level								Projected Population for the Second Quarter of 2015 ^†^							
	Top Three Most Severe Injury (*N* = 2745)		Most Severe Injury (*N* = 1737)		Second Most Severe Injury (*N* = 654)		Third Most Severe Injury (*N* = 354)		Top Three Most Severe Injury (*N* = 1,393,521)		Most Severe Injury (*N* = 879,557)		Second Most Severe Injury (*N* = 332,626)		Third Most Severe Injury (*N* = 181,339)	
	*N*	%	*N*	%	*N*	%	*N*	%	*N*	%	*N*	%	*N*	%	*N*	%
Sprain	656	23.9%	463	26.7%	140	21.4%	53	15.0%	334,754	24.0%	235,974	26.8%	71,163	21.4%	27,618	15.2%
Falls	559	20.4%	422	24.3%	96	14.7%	41	11.6%	277,685	19.9%	208,931	23.8%	47,933	14.4%	20,821	11.5%
Being hit/struck	542	19.7%	303	17.4%	154	23.6%	85	24.0%	272,480	19.6%	152,108	17.3%	77,577	23.3%	42,794	23.6%
Cutting/piercing	428	15.6%	220	12.7%	124	19.0%	84	23.7%	219,913	15.8%	112,624	12.8%	64,034	19.3%	43,256	23.9%
Sport injuries	343	12.5%	199	11.5%	93	14.2%	51	14.4%	178,651	12.8%	103,994	11.8%	48,065	14.5%	26,592	14.7%
Burns/Scald	116	4.2%	70	4.0%	26	4.0%	20	5.7%	57,941	4.2%	34,906	4.0%	13,022	3.9%	10,012	5.5%
Pinch/Crush	49	1.8%	29	1.7%	9	1.4%	11	3.1%	25,295	1.8%	14,851	1.7%	4831	1.5%	5613	3.1%
Animal bite	21	0.8%	7	0.4%	8	1.2%	6	1.7%	10,715	0.8%	3675	0.4%	3965	1.2%	3075	1.7%
Traffic injuries	20	0.7%	18	1.0%	1	0.2%	1	0.3%	10,687	0.8%	9406	1.1%	618	0.2%	663	0.4%
Abrasion	9	0.3%	4	0.2%	3	0.5%	2	0.6%	4260	0.3%	1947	0.2%	1418	0.4%	895	0.5%
Rust of iron powder fall in eye	1	0.0%	1	0.1%	0	0.0%	0	0.0%	570	0.0%	570	0.1%	0	0.0%	0	0.0%
Unknown	1	0.0%	1	0.1%	0	0.0%	0	0.0%	570	0.0%	570	0.1%	0	0.0%	0	0.0%

^†^ Population weighting allocated by age group and gender of survey respondents was applied to sample level to estimate the results with respect to the land-based non-institutional population of Hong Kong in the second quarter of 2015.

**Table 3:** Part of body injured, location of injury, and whether injury was work-related among the most severe injury episode.

		Sample Level		Projected Population for the Second Quarter of 2015 ^‡^	
		All Cause (*N* = 1737)		All Cause (*N* = 879,557)	
		*N*	%	*N*	%
**Part of body(-ies) injured ^†^**					
	Wrist, hand, finger	495	28.5%	251,905	28.6%
	Ankle, foot, toe	421	24.2%	211,487	24.0%
	Knee, lower leg	418	24.1%	211,340	24.0%
	Lower back or lower spine (including waist)	166	9.6%	85,133	9.7%
	Elbow, lower arm	164	9.4%	84,345	9.6%
	Shoulder, upper arm	101	5.8%	51,978	5.9%
	Head (other than eyes & face, but including ears)	84	4.8%	41,579	4.7%
	Thigh	57	3.3%	28,683	3.3%
	Neck	31	1.8%	15,856	1.8%
	Abdomen or pelvis (excluding back and spine)	28	1.6%	13,949	1.6%
	Hip	24	1.4%	11,812	1.3%
	Face, including nose	21	1.2%	10,249	1.2%
	Eye(s)	19	1.1%	9874	1.1%
	Upper back or upper spine	18	1.0%	8653	1.0%
	Chest (excluding back and spine)	15	0.9%	7719	0.9%
	Tooth (teeth)	7	0.4%	3424	0.4%
	Multiple parts	1	0.1%	596	0.1%
**Location where injury was obtained**					
	Home	504	29.0%	250,847	28.5%
	Sport or athletics area	289	16.6%	151,160	17.2%
	Transport area: public highway, street or road	289	16.6%	147,153	16.7%
	Commercial area (non-recreational, e.g., offices)	186	10.7%	95,177	10.8%
	Recreational area, cultural area or public building (e.g., shopping mall, restaurant, park, club house)	139	8.0%	69,749	7.9%
	Industrial or construction area	107	6.2%	52,631	6.0%
	School, educational area	63	3.6%	31,209	3.6%
	Countryside	55	3.2%	27,782	3.2%
	Unspecified place of occurrence	48	2.8%	24,939	2.8%
	Transport area: others (e.g., bus terminal, MTR station, car park)	41	2.4%	21,088	2.4%
	Medical service area	12	0.7%	5904	0.7%
	Residential institution	3	0.2%	1402	0.2%
	Farm or other place of primary production (e.g., livestock farming, fishery)	1	0.1%	515	0.1%
**Work-related injury**					
	Yes	229	13.2%	116,059	13.2%
	No	1508	86.8%	763,498	86.8%

Note: ^†^ Multiple answers were allowed. ^‡^ Population weighting allocated by age group and gender of survey respondents was applied to sample level to estimate the results with respect to the land-based non-institutional population of Hong Kong in the second quarter of 2015.

**Table 4:** Factors associated with unintentional injury by multivariable logistic regression analysis.

		Likelihood of Having Unintentional Injury		
		Odds Ratio	95% CI	*p*-Value
**Employment status (adults)**				
	Employed (office-based/managerial/administrative)	1.608	(1.276,2.026)	<0.001 *
	Employed (manual/physical work)	1.563	(1.235, 1.976)	<0.001 *
	Unemployed	1.119	(0.769,1.628)	0.558
	Retired	Ref		
	Homemaker	1.299	(1.026,1.643)	0.030 *
	Student	1.716	(1.265,2.329)	0.001 *
**Immigrant background**				
	Born in Hong Kong	1.495	(1.114,2.005)	0.007 *
	Immigrant, has lived in Hong Kong for 7 years or more	1.294	(0.954,1.755)	0.098
	Immigrant, has lived in Hong Kong for less than 7 years	Ref		
**Educational background**				
	No schooling/Pre-primary	1.383	(1.034,1.851)	0.029 *
	Primary	1.049	(0.882, 1.247)	0.588
	Secondary	Ref		
	Tertiary	1.175	(1.024,1.347)	0.021 *
**Doctor-diagnosed chronic conditions (No conditions as Ref)**				
	Respiratory disease	1.629	(1.239,2.143)	<0.001 *
	Diseases of the ears/nose/throat	2.200	(1.667,2.902)	<0.001 *
	Liver, stomach and intestinal diseases	1.592	(1.205,2.104)	0.001 *
	Congenital blood diseases	1.582	(1.135,2.205)	0.007 *
	Musculoskeletal diseases	1.314	(1.013, 1.704)	0.040 *
	Skin diseases	1.513	(1.173,1.951)	0.001 *
	Eye disease	1.426	(1.171,1.736)	<0.001 *
	Hearing problem	1.406	(1.020,1.938)	0.037 *
**Alcohol consumption**				
	Never	Ref		
	Ex	1.045	(0.876, 1.247)	0.624
	Non-harmful current ^†^	1.106	(0.959,1.276)	0.167
	Harmful current ^†^	1.516	(1.136,2.023)	0.005 *
**Sleep disturbance ^‡^**				
	No	Ref		
	Yes	1.682	(1.436,1.969)	<0.001 *
**Sleep hour**				
	<6 h	1.383	(1.173,1.631)	<0.001 *
	6–8 h	Ref		
	≥9 h	1.130	(0.907,1.406)	0.276

The results were adjusted for sex, age group, marital status, monthly household income, doctor-diagnosed cancer, stroke, coronary heart diseases, neurological diseases, thyroid disease, kidney diseases, immune diseases, high blood cholesterol, hypertension, diabetes, mental health condition, physical activity level and smoking status, which are insignificant to the likelihood of having an episode of unintentional injury. Notes: * Significant at 0.05 level by multivariable logistic regression, if appropriate. ^†^ Harmful drinking is defined as an Alcohol Use Disorders Identification Test (AUDIT) score ≥8. ^‡^ Sleep disturbance includes having difficulty in falling asleep, maintaining sleep, and early morning awakenings.

**Table 5:** Multivariable logistic regression analysis of factors associated with work-related and sport-related unintentional injury as the most severe unintentional injury episode.

	Likelihood of Work-Related Injury			Likelihood of Sport-Related Injury		
	Odds Ratio	95% CI	*p*-Value	Odds Ratio	95% CI	*p*-Value
**Gender**						
Male				3.192	(2.176,4.684)	<0.001 *
Female				Ref		
**Age group**						
<35				2.758	(1.189,6.397)	0.018*
35–54				2.468	(1.186,5.136)	0.016*
55–74				Ref		
**Employment status (adults)**						
Employed (office-based/managerial/administrative)	Ref			3.171	(1.580,6.364)	0.001 *
Employed (manual/physical work)	2.511	(1.677,3.759)	<0.001 *	Ref		
Unemployed				1.989	(0.563,7.032)	0.286
Retired				1.079	(0.261,4.454)	0.917
Homemaker				1.775	(0.554,5.687)	0.334
Student				8.259	(3.760,18.144)	<0.001 *
**Immigrant background**						
Born in Hong Kong	2.247	(1.536,3.287)	<0.001 *			
Immigrant, has lived in Hong Kong for 7 years or more	3.433	(1.339,8.799)	0.010 *			
Immigrant, has lived in Hong Kong for less than 7 years	Ref					
**Educational background**						
No schooling/Pre-primary	(No observation)					
Primary	2.396	(1.154,4.972)	0.019 *			
Secondary	2.366	(1.502,3.726)	<0.001 *			
Tertiary	Ref					
**Physical activity (sufficient as recommended by WHO)**						
Yes	3.019	(1.572,5.799)	0.001 *	Ref		
No	Ref			2.735	(1.328,5.633)	0.006 *
**Smoking status**						
Never smoker	2.239	(1.275,3.933)	0.005 *	2.326	(1.254,4.312)	0.007 *
Current smoker	1.148	(0.675,1.953)	0.611	Ref		
Former smoker	Ref			2.408	(1.184,4.898)	0.015 *
**Alcohol consumption**						
Never drinker				Ref		
Former drinker				1.371	(0.692,2.718)	0.366
Non-harmful current drinker ^†^				1.417	(0.839,2.392)	0.192
Harmful current drinker ^†^				2.373	(1.050,5.362)	0.038 *

The results were adjusted for marital status, monthly household income, doctor-diagnosed chronic conditions, doctor-diagnosed mental health condition, sleep disturbance and sleep hour, which are insignificant to the likelihood of having an episode of work-related or sport-related unintentional injury. Notes: * Significant at 0.05 level by multivariable logistic regression, if appropriate. ^†^ Harmful drinking is defined as an Alcohol Use Disorders Identification Test (AUDIT) score ≥ 8.

## Data Availability

Restrictions apply to the availability of these data. Data were obtained from Department of Health, the Government of Hong Kong Special Administrative Region.

## Supplementary Materials

The following are available online at https://www.mdpi.com/article/10.3390/ijerph18168826/s1, Table S1: STROBE statement for cross-sectional studies.
