# Supplementary material for: Unintentional Injury Burden in Hong Kong: Results from a Representative Population-Based Survey
Source: Int J Environ Res Public Health. 2021 Aug 21;18(16):8826. doi: 10.3390/ijerph18168826 (PMC8392553; doi:10.3390/ijerph18168826)
Supplement: Supplementary file 1 [file ijerph-18-08826-s001.zip › ijerph-1253853-supplementary.pdf]

Supplementary Material

Supplementary Table S1. Interaction between sleep deprivation/disturbances and alcohol consumption associated with unintentional injury by multivariable logistic regression analysis

Supplementary Table S1. Interaction between sleep deprivation/disturbances and alcohol consumption associated with unintentional injury by multivariable logistic regression analysis

	Likelihood of having unintentional injury		
	Odds ratio	95% CI	P-value
**Interaction of Alcohol consumption and Sleep hour**			
**(Reference: Never drinker or 6-8 hours of sleep)**			
Former drinker × < 6 hours of sleep	1.334	(0.783,2.273)	0.290
Non-harmful current drinker[†] × < 6 hours of sleep	1.341	(0.888,2.026)	0.163
Harmful current drinker[†] × < 6 hours of sleep	1.110	(0.471,2.615)	0.812
Former drinker × ≥ 9 hours of sleep	1.177	(0.647,2.140)	0.594
Non-harmful current drinker[†] × ≥ 9 hours of sleep	0.722	(0.435,1.197)	0.207
Harmful current drinker[†] × ≥ 9 hours of sleep	0.555	(0.171,1.800)	0.327
**Interaction of Alcohol consumption and Any sleep disturbance[‡]**			
**(Reference: Never drinker or Without sleep disturbance)**			
Former drinker × With sleep disturbance	1.157	(0.711,1.881)	0.558
Non-harmful current drinker[†] × With sleep disturbance	1.154	(0.787,1.692)	0.464
Harmful current drinker[†] × With sleep disturbance	0.617	(0.293,1.299)	0.204

The results were adjusted by sex, age group, employment status, marital status, immigrant background, educational background, monthly household income, doctor-diagnosed chronic conditions, mental health condition, physical activity, smoking status, alcohol consumption, sleep disturbance and sleep hour.

Notes:

[†] Harmful drinking is defined as Alcohol Use Disorders Identification Test (AUDIT) scores ≥ 8.

[‡] Sleep disturbance includes having difficulty in falling asleep, maintaining sleep and early morning awakenings.
